# Retraction Note: Patelloplasty in total knee arthroplasty with circumpatellar denervation versus without denervation – a randomized prospective study

**DOI:** 10.1186/s42836-021-00076-6

**Published:** 2021-03-25

**Authors:** S. R. K. Deekshith, K. J. Reddy, R. Raviteja

**Affiliations:** Department of Orthopedics, SVS Medical College and Hospital, Mahabubnagar, Telangana State 500062 India

**Retraction Note: Arthroplasty (2018) 197**

**https://doi.org/10.1186/s42836-021-00076-6**

The Editor-in-Chief has retracted this article because it has been previously published by the same authors [1]. This article is therefore redundant.

The authors have not responded to any correspondence from the editor about this retraction.
